# Analysis of Firearm Violence During the COVID-19 Pandemic in the US

**DOI:** 10.1001/jamanetworkopen.2022.9393

**Published:** 2022-04-28

**Authors:** Shengzhi Sun, Wangnan Cao, Yang Ge, Michael Siegel, Gregory A. Wellenius

**Affiliations:** 1 School of Public Health, Capital Medical University, Beijing, China; 2 Department of Environmental Health, Boston University School of Public Health, Boston, Massachusetts; 3 Department of Social Medicine and Health Education, School of Public Health, Peking University, Beijing, China; 4 Department of Epidemiology and Biostatistics, School of Public Health, University of Georgia, Athens; 5 The Dr. Lynn Cook Hartwig Public Health Program, The University of Southern Mississippi, Hattiesburg; 6 Department of Public Health and Community Medicine, Tufts University School of Medicine, Boston, Massachusetts

## Abstract

**Importance:**

In the US, the COVID-19 pandemic intensified some conditions that may contribute to firearm violence, and a recent surge in firearm sales during the pandemic has been reported. However, patterns of change in firearm violence in the first year of the COVID-19 pandemic in the US remain unclear.

**Objective:**

To quantify the changes in interpersonal firearm violence associated with the pandemic across all 50 US states and the District of Columbia.

**Design, Setting, and Participants:**

This population-based cross-sectional study examined 50 US states and the District of Columbia from January 1, 2016, to February 28, 2021. The COVID-19 pandemic period was defined as between March 1, 2020, and February 28, 2021. Statistical analysis was performed from April to December 2021.

**Main Outcomes and Measures:**

A 2-stage interrupted time-series design was used to examine the excess burden of firearm-related incidents, nonfatal injuries, and deaths associated with the pandemic while accounting for long-term trends and seasonality. In the first stage, separate quasi-Poisson regression models were fit to the daily number of firearm events in each state. In the second stage, estimates were pooled using a multivariate meta-analysis.

**Results:**

In the US (all 50 states and the District of Columbia) during the pandemic period of March 1, 2020, to February 28, 2021, there were 62 485 identified firearm-related incidents, 40 021 firearm-related nonfatal injuries, and 19 818 firearm-related deaths. The pandemic period was associated with 8138 (95% empirical confidence interval [eCI], 2769-12 948) excess incidents (increase of 15.0% [95% eCI, 4.6%-26.1%]), 10 222 (95% eCI, 8284-11 650) excess nonfatal injuries (increase of 34.3% [95% eCI, 26.1%-41.1%]), and 4381 (95% eCI, 2262-6264) excess deaths (increase of 28.4% [95% eCI, 12.9%-46.2%]). The increase in firearm-related violence was more pronounced from June to October 2020 and in Minnesota and New York State.

**Conclusions and Relevance:**

In the US, the first year of the COVID-19 pandemic was associated with an excess burden of firearm-related incidents, nonfatal injuries, and deaths, with substantial temporal and spatial variations.

## Key Points

**Question:**

How did interpersonal firearm violence change temporally and spatially in the first year of the COVID-19 pandemic period in the US?

**Findings:**

In this nationwide cross-sectional study of the US, the pandemic period was associated with a 15.0% increase in firearm-related incidents, a 34.3% increase in firearm-related nonfatal injuries, and a 28.4% increase in firearm-related deaths. The excess burden was more pronounced from June to October 2020 and in Minnesota and New York State.

**Meaning:**

These findings suggest that the COVID-19 pandemic was associated with an excess burden of firearm violence, with substantial temporal and spatial variations.

## Teaser

This cross-sectional study investigates the changes in interpersonal firearm violence associated with the pandemic across all 50 US states and the District of Columbia.

## Introduction

In the US, more than 500 000 deaths were directly attributable to COVID-19 in the first year of the pandemic.^1^ The pandemic and the concomitant public health response profoundly affected nearly every aspect of people’s lives. The impacts on health and well-being of work and school closures and other social distancing measures are only starting to be quantified,^2,3,4,5^ and emerging evidence suggests that pandemic restrictions may have had substantial detrimental effects on population mental health.^3,6^

Worsening economic conditions, psychological strain, and trauma associated with the pandemic, combined with an increase in firearm sales,^7^ could potentially increase the risk of firearm violence in association with the pandemic, thus exacerbating another major public health crisis in the US.^8^ However, only a few studies have examined changes in gun violence associated with the pandemic period,^9,10,11,12,13,14,15,16,17,18^ and most of these were limited to a small number of locations.^10,11,12,13,14,15,16,18^ To our knowledge, no study has quantified the spatial and temporal changes in firearm violence associated with the pandemic at a national scale after controlling for long-term trends and seasonality.

Accordingly, we sought to quantify the change in firearm violence associated with the COVID-19 pandemic by examining nearly 300 000 firearm-related incidents occurring between January 1, 2016, and February 28, 2021, across all 50 US states and the District of Columbia. We used a 2-stage interrupted time-series design, which allowed us to quantify the excess burden of firearm violence associated with the pandemic across the US as a whole and within each state while controlling for long-term trends and seasonality.

## Methods

This cross-sectional followed the Strengthening the Reporting of Observational Studies in Epidemiology (STROBE) reporting guideline. This study did not meet the definition of human subjects research and thus did not require approval by an institutional review board nor informed patient consent, in accordance with 45 CFR §46.

### Firearm Violence Data

We obtained data on firearm violence occurring between January 1, 2016, and February 28, 2021, for the 50 US states and the District of Columbia from the Gun Violence Archive (GVA), a nonprofit organization that compiles real-time information of firearm violence based on daily verified collections from more than 7500 news outlets and other public sources.^19^ Interpersonal firearm violence data collected by GVA include death, injury, or threat with firearm, regardless of intent, but exclude suicides and self-inflicted gunshot wounds. Each firearm-related event in the GVA data has been verified by both initial researchers and secondary validation processes.^20^ Events are then organized into 3 categories: firearm-related incidents, nonfatal injuries, and deaths. For each state, we created a time-series of the number of firearm-related incidents, nonfatal injuries, and deaths per day. The GVA data set has been used for research on firearm violence in the US.^21,22,23^ Data regarding race, gender, and age were not collected because they are not available for download in the GVA data set.

### Statistical Analysis

We used a 2-stage interrupted time-series model^24^ to quantify the time-varying excess burden of firearm violence during the COVID-19 pandemic compared with the prepandemic period, accounting for long-term trends and seasonality. We defined the pandemic period as being from March 1, 2020, to February 28, 2021.

In the first stage of the analyses, we fit a quasi-Poisson time-series regression model to estimate the state-specific relative risk (RR) of firearm violence associated with the pandemic period.^24^ We fit separate models for each state (and the District of Columbia) and for each of the 3 outcomes: the daily number of firearm-related incidents, nonfatal injuries, and deaths. We controlled for long-term trends using a linear term for time, controlled for seasonality using a cyclic cubic B-spline with 3 equal-spaced knots for the day of the year, and included indicator variables for day of the week and federal public holidays in all models. To estimate the excess risk in firearm violence associated with the pandemic, we used a constrained quadratic B-spline for the days from February 15, 2020, to February 28, 2021. The number of knots for the constrained quadratic B-spline were guided by the minimum quasi–Akaike information criterion and were placed equally on the pandemic period (eTable 1 in the Supplement).^25^ This function constrains the excess risk to start from null on February 15, 2020, and then allows it to vary flexibly until the end of the study period. In sensitivity analyses, we varied the key model parameters to assess the robustness of our findings.

In the second stage of the analyses we used a random-effects meta-analytic model to pool the estimates of the state-specific associations of the pandemic period with firearm violence.^26^ We used the fitted meta-analytical model to derive the best linear unbiased predictions (BLUP) of the association in each state.^26^ The BLUP represents a trade-off between the state-specific association provided by the first stage regression and the pooled association.^26^ This approach allows states with small daily counts to borrow information from larger populations that share similar characteristics.^26^ In each state, we used the RR corresponding to each day of the pandemic to calculate the excess number and percentage excess events.

#### Quantification of Excess Burden

The model-estimated daily number of excess firearm-related incidents, nonfatal injuries, and deaths for each state was estimated as [(RR − 1)/RR] × *n*, where *n* is the daily observed number of firearm-related incidents, nonfatal injuries, and deaths during the pandemic period, and RR is the relative risk corresponding to each day of the pandemic as estimated from the BLUPs.^27^ We calculated the relative excess in firearm violence events over the pandemic period as the sum of the model-estimated excess number of firearm events divided by the sum of the expected number of firearm events over the pandemic period. The sum of the expected number of firearm events was calculated as the sum of the number of observed firearm events minus the sum of the model-estimated excess number of firearm events over the pandemic period. We estimated the 95% empirical confidence intervals using Monte Carlo simulation (n = 5000) assuming a multivariate normal distribution of the coefficients in the BLUP.^27^

#### Comparison With Data From Philadelphia

As a point of comparison, we obtained publicly available data on firearm violence from the Philadelphia Police Department registry of shooting victims.^28^ We repeated the 2-stage analysis described previously to estimate the excess firearm burden associated with the first year of the pandemic in Philadelphia comparing the results using data from GVA vs Philadelphia Police Department registry of shooting victims.

We conducted all statistical analyses from April to December 2021 with R software version 4.0.3 (R Project for Statistical Computing), using the mgcv and dlnm package for fitting the 2-stage interrupted time-series regression and the mvmeta package for performing random-effect meta-analytic models. A 2-sided *P* < .05 was considered to indicate statistical significance.

## Results

### Descriptive Statistics

Nationally in the US (all 50 states and the District of Columbia) between January 1, 2016, and February 28, 2021, there were 295 280 documented firearm-related incidents, 165 335 nonfatal injuries, and 83 491 deaths, corresponding to an annual mean of 57 151 (17.5 per 100 000 population) firearm-related incidents, 32 000 (9.8 per 100 000 population) firearm-related nonfatal injuries, and 16 160 (4.9 per 100 000 population) firearm-related deaths. The District of Columbia experienced the highest rate of firearm-related incidents (98.1 per 100 000 population), nonfatal injuries (60.9 per 100 000 population), and deaths (19.2 per 100 000 population), followed by Louisiana for firearm-related incidents and deaths or Illinois for firearm-related nonfatal injuries (eTable 2 in the Supplement).

**Figure 1.**
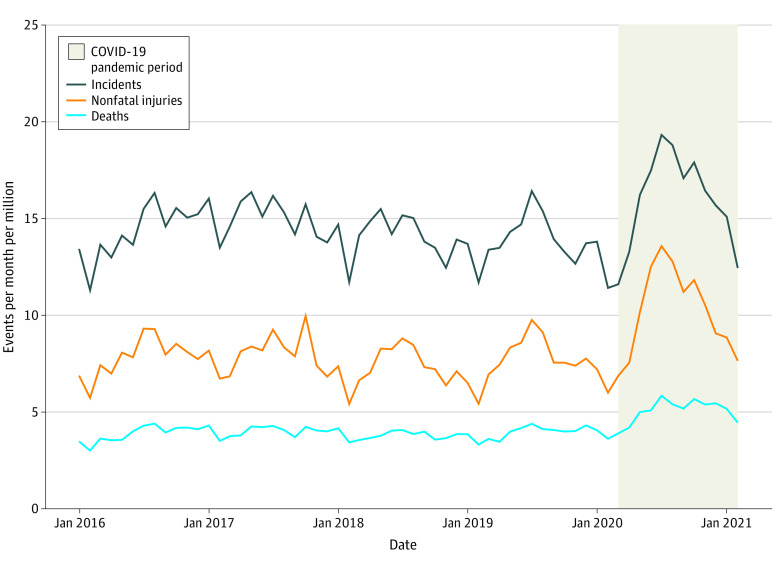
Firearm-Related Events Per Month Per Million People, January 1, 2018, to February 28, 2021, United States

The rate of firearm violence appeared to increase from February 15, 2020, into the pandemic period (Figure 1). Compared with the baseline period, the rate of firearm violence was higher during the pandemic period nationally and in several states, with the District of Columbia having the greatest number of incidents, nonfatal injuries, and deaths; and New York State and Minnesota exhibiting the largest relative increase (eFigure 1 in the Supplement).

### Excess Burden in Firearm Violence During the Pandemic Period

**Figure 2.**
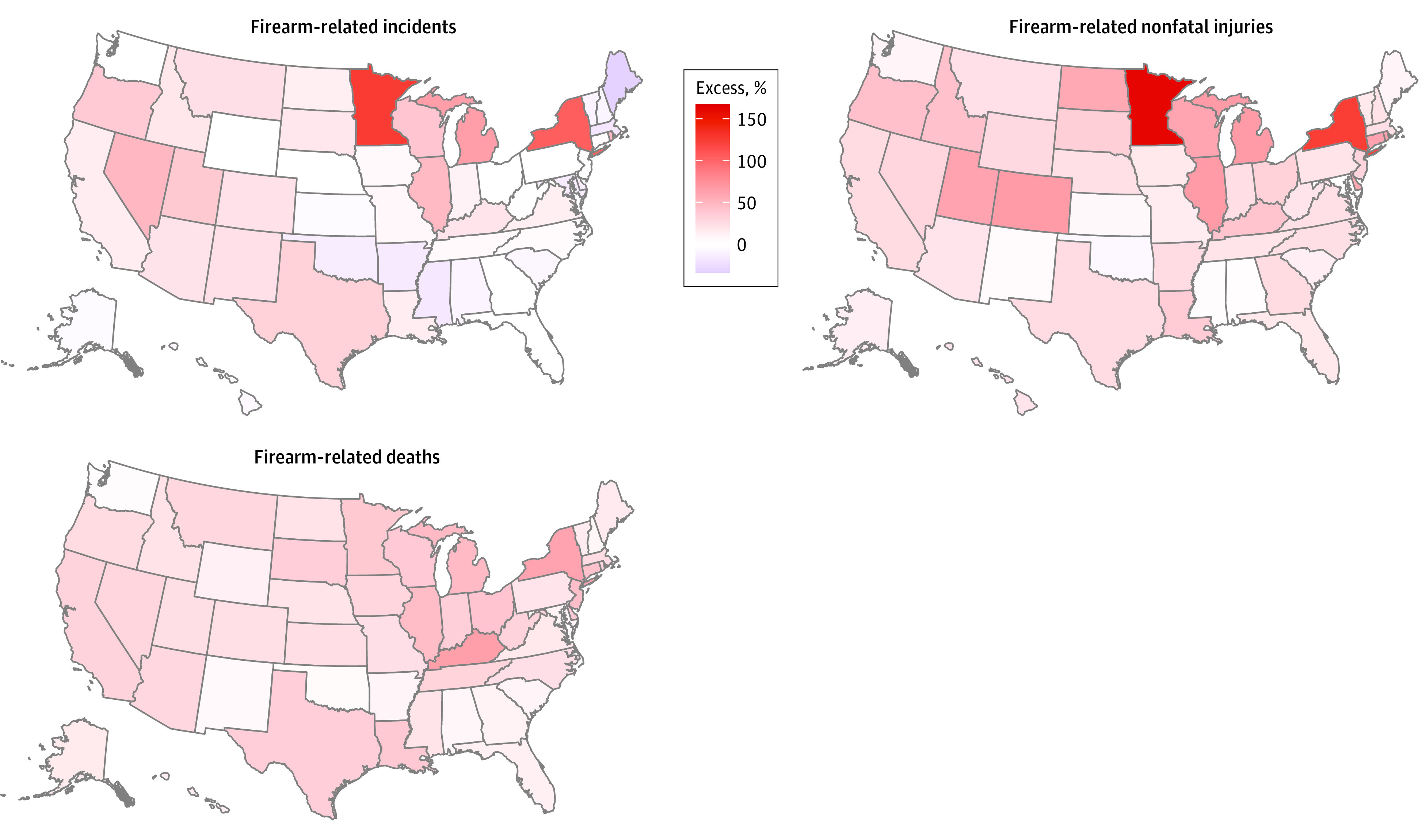
Maps of Excess Percentage of Firearm-Related Events During the COVID-19 Pandemic by State in the United States For corresponding excess percentages for each state, refer to the Table.

During the pandemic period of March 1, 2020, to February 28, 2021, there were 62 485 identified firearm-related incidents, 40 021 firearm-related nonfatal injuries, and 19 818 firearm-related deaths. The pandemic period was associated with an additional 8138 (95% eCI, 2769-12 948) firearm-related incidents compared with the baseline period, corresponding to a relative increase of 15.0% (95% eCI, 4.6%-26.1%) (Table). The excess burden of firearm-related incidents exhibited pronounced geographic variation (Figure 2 and Table), with the greatest absolute number increase observed in New York State (1784 [95% eCI, 1607-1949]), Illinois (1673 [95% eCI, 1360-1966]), Texas (1244 [95% eCI, 900-1559]), and Michigan (1099 [95% eCI, 919-1266]). These 4 states accounted for 71.3% of all the excess firearm-related incidents associated with the pandemic in the US (Table). In relative terms, Minnesota experienced the greatest increase in firearm-related incidents (119.0% [95% eCI [86.7%-156.8%]), followed by New York State (104.1% [95% eCI, 84.9%-125.8%]) (Table).

**Table.** Firearm Violence and Estimated Excess During the COVID-19 Period in 50 US States and the District of Columbia^a^

Jurisdiction	Firearm-related incidents			Firearm-related nonfatal injuries			Firearm-related deaths		
	Total	Excess (95% eCI)		Total	Excess (95% eCI)		Total	Excess (95% eCI)	
		No.	%		No.	%		No.	%
Alabama	1221	−130 (−287 to 12)	−9.6 (−19.0 to 1.0)	739	25 (−82 to 109)	3.4 (−10.0 to 17.2)	514	41 (−26 to 99)	8.7 (−4.8 to 23.9)
Alaska	101	−6 (−36 to 16)	−5.6 (−26.2 to 19.5)	42	5 (−7 to 13)	14.5 (−13.5 to 46.8)	36	6 (−3 to 13)	19.1 (−8.5 to 54.2)
Arizona	863	151 (50 to 240)	21.2 (6.1 to 38.6)	394	68 (5 to 117)	20.8 (1.2 to 42.1)	402	93 (42 to 136)	29.9 (11.8 to 51.1)
Arkansas	639	−131 (−246 to −35)	−17.0 (−27.8 to −5.2)	410	85 (21 to 135)	26.1 (5.5 to 49.0)	250	24 (−17 to 59)	10.7 (−6.4 to 30.8)
California	3807	518 (206 to 793)	15.7 (5.7 to 26.3)	2430	514 (297 to 696)	26.8 (13.9 to 40.2)	1722	424 (299 to 540)	32.7 (21.0 to 45.6)
Colorado	877	157 (52 to 237)	21.8 (6.3 to 37.1)	528	215 (154 to 261)	68.8 (41.4 to 97.6)	279	54 (13 to 88)	23.7 (4.7 to 46.0)
Connecticut	813	38 (−69 to 131)	4.9 (−7.8 to 19.1)	521	202 (143 to 249)	63.2 (38.0 to 91.3)	121	38 (18 to 54)	45.0 (17.2 to 80.6)
Delaware	375	−63 (−141 to 3)	−14.4 (−27.3 to 0.8)	250	93 (58 to 121)	59.4 (30.0 to 93.1)	70	21 (7 to 32)	43.0 (11.2 to 85.5)
District of Columbia	1052	123 (−8 to 236)	13.3 (−0.8 to 29.0)	641	177 (94 to 245)	38.2 (17.2 to 61.8)	186	28 (−4 to 54)	17.4 (−2.4 to 41)
Florida	2757	−38 (−319 to 219)	−1.4 (−10.4 to 8.6)	1744	274 (98 to 417)	18.7 (5.9 to 31.4)	1139	139 (21 to 245)	13.8 (1.9 to 27.5)
Georgia	1703	−10 (−197 to 164)	−0.6 (−10.4 to 10.7)	1092	229 (111 to 331)	26.6 (11.4 to 43.5)	779	81 (−6 to 159)	11.6 (−0.7 to 25.6)
Hawaii	57	−4 (−27 to 12)	−7.0 (−32.2 to 27.4)	23	4 (−5 to 9)	19.6 (−17.1 to 69.4)	13	2 (−2 to 4)	13.7 (−14.7 to 50.8)
Idaho	126	21 (−8 to 44)	20.2 (−5.8 to 53.8)	43	15 (4 to 22)	51.0 (9.1 to 102.9)	48	9 (−2 to 18)	24.5 (−3.7 to 60.0)
Illinois	5076	1673 (1360 to 1966)	49.2 (36.6 to 63.2)	4251	1724 (1479 to 1953)	68.2 (53.3 to 85.0)	1122	357 (273 to 433)	46.7 (32.2 to 62.8)
Indiana	1554	153 (−8 to 302)	10.9 (−0.5 to 24.1)	1027	199 (84 to 288)	24.1 (8.9 to 38.9)	541	141 (83 to 193)	35.4 (18.2 to 55.3)
Iowa	589	35 (−53 to 108)	6.3 (−8.3 to 22.6)	225	34 (−7 to 66)	18.0 (−3.0 to 41.8)	96	22 (5 to 36)	29.6 (5.3 to 60.5)
Kansas	498	−35 (−119 to 37)	−6.6 (−19.3 to 8.0)	243	17 (−35 to 58)	7.4 (−12.6 to 31.3)	175	35 (7 to 60)	25.4 (4.3 to 51.6)
Kentucky	1197	203 (82 to 316)	20.4 (7.3 to 35.8)	684	203 (125 to 262)	42.2 (22.3 to 62.2)	417	168 (128 to 203)	67.6 (44.0 to 95.1)
Louisiana	1895	254 (83 to 413)	15.5 (4.6 to 27.9)	1442	390 (256 to 506)	37.1 (21.6 to 54.0)	739	206 (136 to 269)	38.6 (22.6 to 57.1)
Maine	91	−46 (−84 to −16)	−33.7 (−48.1 to −15.3)	31	3 (−6 to 10)	11.2 (−16.6 to 47.7)	25	4 (−3 to 9)	17.1 (−10.7 to 51.9)
Maryland	1610	−323 (−526 to −135)	−16.7 (−24.6 to −7.8)	1090	−12 (−159 to 115)	−1.1 (−12.7 to 11.8)	525	54 (−10 to 111)	11.6 (−1.8 to 26.9)
Massachusetts	897	−230 (−375 to −100)	−20.4 (−29.5 to −10.0)	380	73 (19 to 117)	23.9 (5.3 to 44.5)	132	29 (6 to 48)	27.9 (4.9 to 56.7)
Michigan	2752	1099 (919 to 1266)	66.5 (50.1 to 85.2)	1766	707 (565 to 816)	66.8 (47.1 to 86.0)	662	220 (158 to 276)	49.9 (31.3 to 71.7)
Minnesota	1156	628 (537 to 706)	119.0 (86.7 to 156.8)	650	400 (347 to 441)	159.5 (114.7 to 211.0)	189	55 (26 to 79)	41.1 (16.2 to 71.4)
Mississippi	860	−188 (−327 to −69)	−17.9 (−27.6 to −7.4)	532	22 (−67 to 89)	4.4 (−11.2 to 20.0)	396	70 (19 to 115)	21.6 (5.2 to 40.9)
Missouri	1775	136 (−46 to 308)	8.3 (−2.5 to 21.0)	1118	154 (14 to 260)	16.0 (1.2 to 30.3)	752	145 (66 to 216)	24.0 (9.7 to 40.3)
Montana	116	23 (−4 to 43)	24.3 (−3.2 to 59.4)	44	9 (−6 to 19)	25.5 (−11.2 to 78.0)	50	12 (1 to 20)	30.5 (1.6 to 66.4)
Nebraska	446	2 (−76 to 67)	0.4 (−14.6 to 17.6)	171	33 (0 to 60)	24.1 (−0.2 to 54.3)	63	11 (−1 to 22)	22.2 (−2.2 to 54.2)
Nevada	483	161 (104 to 208)	50.1 (27.5 to 75.3)	228	55 (−25 to 108)	31.8 (−9.8 to 89.7)	206	48 (10 to 79)	30.5 (5.3 to 62.1)
New Hampshire	118	−13 (−48 to 14)	−10.1 (−28.8 to 13.6)	32	6 (−5 to 13)	21.2 (−13.3 to 64.9)	18	2 (−3 to 6)	10.3 (−16.1 to 44.7)
New Jersey	1169	−38 (−187 to 98)	−3.1 (−13.8 to 9.2)	799	183 (85 to 263)	29.7 (11.8 to 48.9)	269	82 (48 to 110)	43.8 (21.9 to 68.8)
New Mexico	511	97 (25 to 158)	23.5 (5.2 to 44.7)	180	10 (−30 to 44)	5.9 (−14.3 to 31.9)	174	11 (−23 to 39)	6.9 (−11.6 to 29.3)
New York	3498	1784 (1607 to 1949)	104.1 (84.9 to 125.8)	2316	277 (1147 to 1395)	122.9 (98.1 to 151.4)	598	230 (177 to 277)	62.6 (41.9 to 86.4)
North Carolina	1981	98 (−94 to 280)	5.2 (−4.5 to 16.4)	1312	254 (126 to 360)	24.0 (10.6 to 37.8)	749	148 (74 to 216)	24.7 (11.0 to 40.5)
North Dakota	105	10 (−12 to 27)	14.9 (−12.9 to 51.5)	33	12 (3 to 18)	57.4 (11.2 to 123.1)	16	3 (−1 to 6)	21.2 (−4.3 to 55.2)
Ohio	2629	−30 (−307 to 227)	−1.1 (−10.4 to 9.4)	1834	465 (292 to 600)	33.9 (19.0 to 48.6)	871	259 (185 to 326)	42.3 (27.0 to 59.9)
Oklahoma	676	−125 (−237 to −28)	−15.7 (−26.0 to −4.0)	358	−31 (−104 to 24)	−8.0 (−22.4 to 7.1)	258	14 (−30 to 52)	5.9 (−10.5 to 25.4)
Oregon	519	144 (80 to 198)	38.5 (18.2 to 61.8)	273	87 (45 to 120)	46.7 (19.9 to 78.2)	142	30 (6 to 51)	27.1 (4.3 to 56.0)
Pennsylvania	3204	58 (−249 to 344)	1.9 (−7.2 to 12.0)	2663	458 (210 to 659)	20.8 (8.6 to 32.9)	808	141 (58 to 216)	21.1 (7.7 to 36.6)
Rhode Island	131	44 (20 to 64)	50.3 (17.5 to 95.3)	56	23 (9 to 32)	67.5 (19.8 to 136.5)	29	7 (0 to 12)	31.2 (−0.6 to 71.9)
South Carolina	1402	−145 (−318 to 11)	−9.4 (−18.5 to 0.8)	923	117 (4 to 211)	14.5 (0.4 to 29.6)	523	50 (−19 to 109)	10.5 (−3.5 to 26.4)
South Dakota	105	17 (−9 to 37)	19.3 (−8.2 to 53.8)	32	10 (1 to 16)	44.1 (1.6 to 103.6)	22	6 (0 to 10)	35.8 (1.7 to 81.5)
Tennessee	1801	104 (−83 to 273)	6.1 (−4.4 to 17.9)	1178	196 (69 to 298)	19.9 (6.2 to 33.9)	705	171 (102 to 233)	31.9 (16.9 to 49.5)
Texas	4849	1244 (900 to 1559)	34.5 (22.8 to 47.4)	2537	533 (314 to 710)	26.6 (14.1 to 38.8)	1808	482 (342 to 608)	36.3 (23.3 to 50.6)
Utah	322	92 (45 to 131)	39.9 (16.4 to 68.7)	130	50 (26 to 68)	62.2 (25.1 to 109.4)	83	16 (0 to 29)	23.6 (−0.3 to 54.4)
Vermont	63	−8 (−31 to 9)	−11.6 (−33.3 to 17.6)	18	2 (−2 to 5)	15.6 (−9.4 to 43)	13	2 (−1 to 4)	17.7 (−6.3 to 47.8)
Virginia	1561	209 (59 to 349)	15.4 (3.9 to 28.8)	1056	196 (83 to 288)	22.8 (8.6 to 37.4)	469	74 (15 to 125)	18.7 (3.2 to 36.5)
Washington	551	−8 (−94 to 65)	−1.5 (−14.6 to 13.5)	326	28 (−31 to 74)	9.4 (−8.6 to 29.2)	210	9 (−28 to 40)	4.6 (−11.6 to 23.5)
West Virginia	304	−12 (−73 to 37)	−3.9 (−19.3 to 14.0)	146	25 (−4 to 48)	20.8 (−2.5 to 49.4)	113	28 (8 to 44)	32.5 (7.6 to 64.6)
Wisconsin	1598	447 (309 to 577)	38.8 (23.9 to 56.4)	1071	402 (305 to 478)	60.1 (39.8 to 80.7)	277	78 (42 to 109)	39.5 (17.8 to 64.7)
Wyoming	27	0 (−12 to 8)	−1.6 (−30.0 to 38.5)	9	2 (−2 to 4)	26.9 (−18.9 to 95.2)	14	2 (−1 to 4)	13.9 (−6.5 to 37.8)
United States	62 485	8138 (2769 to 12 948)	15.0 (4.6 to 26.1)	40 021	10 222 (8284 to 11 650)	34.3 (26.1 to 41.1)	19 818	4381 (2262 to 6264)	28.4 (12.9 to 46.2)

Abbreviation: eCI, empirical confidence interval.

^a^ The COVID-19 period was defined as March 1, 2020, to February 28, 2021.

There were an estimated 10 222 (95% eCI [8284-11 650]) excess firearm-related nonfatal injuries and 4381 (95% eCI, 2262-6264) excess firearm-related deaths associated with the pandemic, corresponding to 34.3% (95% eCI, 26.1%-41.1%) and 28.4% (95% eCI, 12.9%-46.2%) excess, respectively (Table). The patterns of geographic variations in excess burden for firearm-related nonfatal injuries and deaths were similar to those for firearm-related incidents (Table). Excess firearm-related deaths occurred in all 50 states, with the greatest relative increases observed in Kentucky (67.6% [95% ECI, 44.0%-95.1%]) and New York (62.6% [95% eCI, 41.9%-86.4%]) (Table). In sensitivity analyses using different spline functions or degrees of freedom for the days from February 15, 2020, to February 28, 2021, results were not materially different (eTable 3 and eFigure 2 in the Supplement).

### Temporal Trends in the Pandemic-Associated Excess Risk of Firearm Violence

**Figure 3.**
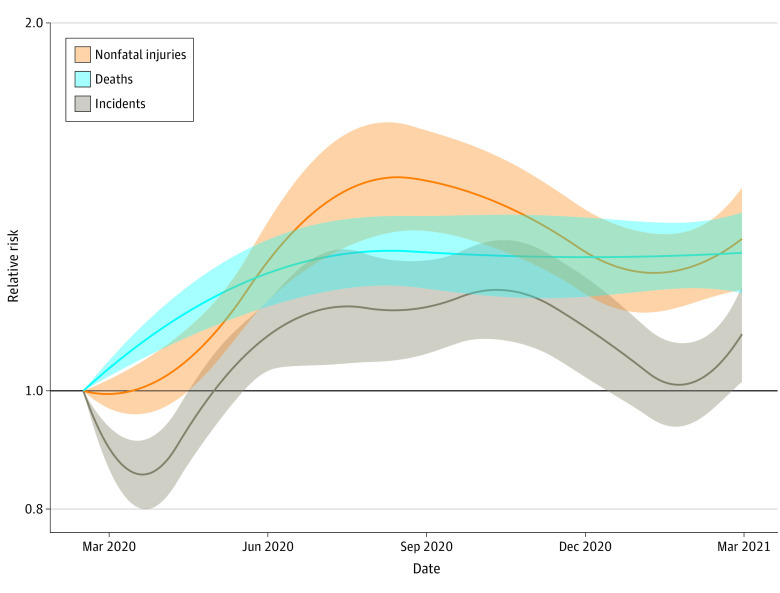
Temporal Trend in Excess Risk Associated With the COVID-19 Pandemic Period in the United States The bands are the 95% empirical confidence interval for the excess risk in the United States.

Nationally, the onset of the pandemic was associated with an initial decline in firearm-related incidents, reaching a nadir around April 2020, followed by an increase in incidents through October 2020 (Figure 3). An initial decline coincident with the onset of the pandemic was not evident in firearm-related deaths or injuries, but all 3 types of firearm violence peaked near July 2020. These temporal patterns were similar across many states (eFigure 3 in the Supplement).

### Comparison With Data From Philadelphia

The number of firearm-related deaths reported in Philadelphia were very similar in the GVA data and the Philadelphia Police Department registry of shooting victims, with somewhat larger differences between these sources for reported firearm-related incidents and nonfatal injuries (eTable 4 in the Supplement). Specifically, the GVA reported 2.3% (10 of 442) more firearm-related deaths, 14.1% (327 of 2324) fewer firearm-related incidents, and 16.8% (406 of 2410) fewer firearm-related nonfatal injuries compared with the registry. The difference in the number of events reported between the 2 data sets was approximately constant over time for firearm-related deaths. There appears to be variation over time for firearm-related incidents and nonfatal injuries, with slightly larger differences through 2018 (eFigure 4 and eFigure 5 in the Supplement).

Using these 2 sources of data, the estimated firearm burden associated with the pandemic in Philadelphia was also similar for firearm-related deaths, with somewhat larger differences for firearm-related incidents and nonfatal injuries (eTable 4 and eFigure 6 in the Supplement). For example, we observed that the pandemic period was associated with a 25.0% (95% eCI, 5.3%-45.4%) increase in firearm-related deaths using the Philadelphia Police Department’s registry of shooting victims and 24.8% (95% eCI, 5.0%-45.4%) using the GVA data. For firearm-related incidents, the estimates were 51.5% (95% eCI, 37.6%-65.5%) using the Philadelphia Police Department’s registry of shooting victims and 15.2% (95% eCI, 5.2%-25.6%) using the GVA data.

## Discussion

Leveraging data from nearly 300 000 firearm-related incidents across the 50 US states and the District of Columbia between January 2016 and February 2021, we evaluated changes in the burden of firearm violence associated with the COVID-19 pandemic across the US. Nationally, we found that the first year of the COVID-19 pandemic was associated with more than 8000 excess firearm incidents across the country, reflecting a relative increase of 15%. The first year of the pandemic was also associated with a 34% increase in firearm-related injuries and a 28% increase in firearm-related deaths, with substantial variation across states. The increase in firearm incidents was more pronounced from June to October 2020 and in Minnesota and New York State.

Our firearm-related mortality findings are consistent with national data from the Centers for Disease Control and Prevention based on the National Center for Health Statistics vital statistics program, which reveal an increase in the age-adjusted, national firearm-related homicide rate from 4.7 per 100 000 in 2019 to 6.4 per 100 000 in 2020, a 34% increase.^29^ These data also reveal that the 2 states with the largest percentage increases in firearm-related homicide rates from 2019 to 2020 were New York (74%) and Kentucky (70%), comparable to what we found.^29^

Only a few prior studies have examined the changes in firearm violence associated with the COVID-19 pandemic.^9,10,11,12,13,14,15,16,17^ Our finding of excess firearm incidents associated with the pandemic is consistent with localized findings from most prior studies.^9,11,12,13^ For example, using data from city police departments, as of April 4, 2020, Sutherland et al^12^ found shooting incidents increased 18.6% in New York, 6.0% in Chicago, and 10.3% in Los Angeles compared with 2018, respectively. We are not aware of prior studies that have quantified the excess burden of firearm violence on a national scale controlling for long-term and seasonal trends.

We observed that the first year of pandemic was associated with a particularly large excess firearm burden in a few states, including Minnesota and New York. We are not aware of prior studies that systematically examined the spatial heterogeneity of firearm violence associated with the pandemic. Our findings are consistent with a cross-sectional analysis comparing firearm violence in 2020 up to April 4 to 2018 in New York City, Chicago, Baltimore, and Los Angeles, which found that New York City witnessed the highest increase compared with the other cities in shooting incidents.^12^ Although the spatial pattern of results is potentially interesting, the current study is not designed or able to explain the causes of heterogeneity across states.

We found that within the first year of the pandemic, the excess burden of firearm violence peaked between June and October 2020. This finding is consistent with an analysis of patients presenting with violent penetrating injuries to the emergency department of a large hospital in Boston, which noted that excess firearm injuries peaked in July 2020.^18^

We found that the pandemic period was associated with an initial small decrease in the rates of firearm-related incidents, coincident with the period when stay-at-home orders were implemented in many US states. However, similar decreases were not evident in the rates of firearm-related nonfatal injuries or deaths, raising the possibility that the apparent reduced risk in firearm-related events early in the pandemic period might reflect a temporary reporting artifact. Although it is not directly comparable, a study in Japan reported an increase in suicide during the pandemic following an initial decline at the beginning of the pandemic in Japan.^4^

The broad public health importance of our findings is that the COVID-19 pandemic affected population health far beyond the direct morbidity and mortality caused by infection with the novel coronavirus itself. The public health consequences of the pandemic also included a significant increase in firearm-related morbidity and mortality. This may in part explain the finding that the United States experienced much greater excess all-cause mortality than other high-mortality countries.^30^ Future research should attempt to identify the factors that are associated with geographical differences in the excess all-cause mortality, including excess firearm-related mortality, associated with the COVID-19 pandemic.

### Limitations and Strengths

These results need to be interpreted in light of several important limitations. First, although we controlled for long-term trends, seasonality, day of the week, and federal holidays, we cannot distinguish the higher excess burden of firearm violence from other changes that simultaneously occurred during the pandemic period, such as civic unrest related to police violence and racism. Thus, we cannot interpret that the higher excess burden of firearm violence is solely attributable to the pandemic or pandemic-era restrictions. Second, the completeness and quality of the GVA data may vary over space and/or time, especially for nonfatal injuries and incidents. Comparison with an independent data source for the city of Philadelphia suggests that the GVA data may differ somewhat from official statistics, especially for firearm-related incidents and nonfatal injuries. However, Philadelphia may or may not be representative of the quality of the GVA data in other locations. Future studies would benefit from additional validation of the GVA data, particularly for reported firearm-related incidents and nonfatal injuries. Third, we did not examine the excess burden of firearm violence by types of deaths (eg, homicide or unintentional shootings) or by personal characteristics (eg, age or race, gender) as GVA does not publicly provide this information.

On the other hand, to our knowledge, this is the first nationwide study to quantify the excess burden in firearm violence during the COVID-19 pandemic in the US as a whole and in each state, and it is the first to examine the temporal changes in the excess burden. Findings of this study contribute to a more comprehensive assessment of the association of the COVID-19 pandemic with population health, well-being, and behaviors.

## Conclusions

In this nationwide study, the first year of the COVID-19 pandemic was associated with a higher excess burden of firearm-related incidents, nonfatal injuries, and deaths in the US. The excess burden of firearm violence was more pronounced from June to October 2020 and in Minnesota and New York State.

## Notes

Supplement. **eTable 1.** The Quasi Akaike’s Information Criterion by the Number of Knots for the Constrained Quadratic B-Spline for the Days From February 15, 2020, to February 28, 2021 **eTable 2.** The Annual Average Number of Firearm-Related Incidents, Non-fatal Injuries, and Deaths During the Study Period of 1 January 2016 to 28 February 2021 by State in the United States **eTable 3.** Percent Excess Firearm Violence During the COVID-19 Pandemic in the 50 US States and the District of Columbia Using Different Spline Functions and Degrees of Freedom (dfs) for the Days From February 15, 2020, to February 28, 2021 **eTable 4.** Number of Firearm Violence and Estimated Excess Number and Percent Excess During the Coronavirus Disease 2019 Period (i.e., 1 March 2020 to 28 February 2021) in City of Philadelphia Using Data from Philadelphia Police Department and Gun Violence Archive (GVA) **eFigure 1.** The Number of Firearm-Related Events per Month per Million People Between January 1, 2018 and February 28, 2021 by State in the United States **eFigure 2.** Temporal Trend in Excess Risk Associated With the COVID-19 Pandemic Period Using A Quadratic B-Spline (Main Model) and a Natural Spline With 3 Degrees of Freedom for the Days From February 15, 2020, to February 28, 2021 in the United States **eFigure 3.** Temporal Trend in Excess Risk by State Associated With the COVID-19 Pandemic Period by State in the United States **eFigure 4.** The Number Difference of Firearm-Related Events Between Data From Philadelphia Police Department and Gun Violence Archive (GVA) **eFigure 5.** The Ratio of Number of Non-fatal to Fatal Incidents in GVA, January 1, 2016 to February 28, 2021 **eFigure 6.** Temporal Trend in Excess Risk in Firearm-related Incidents (A), Non-fatal Injuries (B), and Deaths (C) During the Pandemic Period in the City of Philadelphia Using Data From Gun Violence Archive (GVA) and Philadelphia Police Department (PPD)
eReferences
