# Supplementary material for: Analysis of Firearm Violence During the COVID-19 Pandemic in the US
Source: JAMA Netw Open. 2022 Apr 28;5(4):e229393. doi: 10.1001/jamanetworkopen.2022.9393 (PMC9051986; doi:10.1001/jamanetworkopen.2022.9393)
Supplement: Supplement. — eTable 1. The Quasi Akaike’s Information Criterion by the Number of Knots for the Constrained Quadratic B-Spline for the Days From February 15, 2020, to February 28, 2021 eTable 2. The Annual Average Number of Firearm-Related Incidents, Non-fatal Injuries, and Deaths During the Study Period of 1 January 2016 to 28 February 2021 by State in the United States eTable 3. Percent Excess Firearm Violence During the COVID-19 Pandemic in the 50 US States and the District of Columbia Using Different Spline Functions and Degrees of Freedom (dfs) for the Days From February 15, 2020, to February 28, 2021 eTable 4. Number of Firearm Violence and Estimated Excess Number and Percent Excess During the Coronavirus Disease 2019 Period (i.e., 1 March 2020 to 28 February 2021) in City of Philadelphia Using Data from Philadelphia Police Department and Gun Violence Archive (GVA) eFigure 1. The Number of Firearm-Related Events per Month per Million People Between January 1, 2018 and February 28, 2021 by State in the United States eFigure 2. Temporal Trend in Excess Risk Associated With the COVID-19 Pandemic Period Using A Quadratic B-Spline (Main Model) and a Natural Spline With 3 Degrees of Freedom for the Days From February 15, 2020, to February 28, 2021 in the United States eFigure 3. Temporal Trend in Excess Risk by State Associated With the COVID-19 Pandemic Period by State in the United States eFigure 4. The Number Difference of Firearm-Related Events Between Data From Philadelphia Police Department and Gun Violence Archive (GVA) eFigure 5. The Ratio of Number of Non-fatal to Fatal Incidents in GVA, January 1, 2016 to February 28, 2021 eFigure 6. Temporal Trend in Excess Risk in Firearm-related Incidents (A), Non-fatal Injuries (B), and Deaths (C) During the Pandemic Period in the City of Philadelphia Using Data From Gun Violence Archive (GVA) and Philadelphia Police Department (PPD) eReferences [file jamanetwopen-e229393-s001.pdf]

Supplemental Online Content

Sun S, Cao W, Ge Y, Siegel M, Wellenius GA. Analysis of firearm violence during the COVID-19 pandemic in the United States. *JAMA Netw Open.* 2022;5(4):e229393. doi:10.1001/jamanetworkopen.2022.9393

eTable 1. The Quasi Akaike's Information Criterion by the Number of Knots for the Constrained Quadratic B-Spline for the Days From February 15, 2020, to February 28, 2021

eTable 2. The Annual Average Number of Firearm-Related Incidents, Non-fatal Injuries, and Deaths During the Study Period of 1 January 2016 to 28 February 2021 by State in the United States

eTable 3. Percent Excess Firearm Violence During the COVID-19 Pandemic in the 50 US States and the District of Columbia Using Different Spline Functions and Degrees of Freedom (dfs) for the Days From February 15, 2020, to February 28, 2021

eTable 4. Number of Firearm Violence and Estimated Excess Number and Percent Excess During the Coronavirus Disease 2019 Period (i.e., 1 March 2020 to 28 February 2021) in City of Philadelphia Using Data from Philadelphia Police Department and Gun Violence Archive (GVA)

eFigure 1. The Number of Firearm-Related Events per Month per Million People Between January 1, 2018 and February 28, 2021 by State in the United States

eFigure 2. Temporal Trend in Excess Risk Associated With the COVID-19 Pandemic Period Using A Quadratic B-Spline (Main Model) and a Natural Spline With 3 Degrees of Freedom for the Days From February 15, 2020, to February 28, 2021 in the United States

eFigure 3. Temporal Trend in Excess Risk by State Associated With the COVID-19 Pandemic Period by State in the United States

eFigure 4. The Number Difference of Firearm-Related Events Between Data From Philadelphia Police Department and Gun Violence Archive (GVA)

eFigure 5. The Ratio of Number of Non-fatal to Fatal Incidents in GVA, January 1, 2016 to February 28, 2021

eFigure 6. Temporal Trend in Excess Risk in Firearm-related Incidents (A), Non-fatal Injuries (B), and Deaths (C) During the Pandemic Period in the City of Philadelphia

© 2022 Sun S et al. *JAMA Network Open.*

Using Data From Gun Violence Archive (GVA) and Philadelphia Police Department (PPD)

eReferences

This supplemental material has been provided by the authors to give readers additional information about their work.

© 2022 Sun S et al. *JAMA Network Open.*

eTable 1. The Quasi Akaike's Information Criterion by the number of knots for the constrained quadratic B-spline for the days from February 15, 2020, to February 28, 2021.

Number of knots	Firearm-related incidents	Firearm-related non-fatal injuries	Firearm-related deaths
1	330889.8	277604	**200134.5**
2	330724.2	277605	200202.4
3	330562.4	**277570.2**	200261.9
4	330563.1	277605.3	200328.6
5	330578.8	277753.6	200381.2
6	**330551.5**	277776.3	200415.3
7	330613.8	277859.1	200473
8	330649.9	277947.6	200524.3
9	330692.5	278063.3	200601.9
10	330725.7	278115.8	200669.9
11	330723	278145.1	200698.4
12	330739.8	278223.4	200776.6
13	330836	278365.3	200800.9
14	330885.4	278460.5	200834.8

© 2022 Sun S et al. *JAMA Network Open.*

eTable 2. The annual average number of firearm-related incidents, non-fatal injuries, and deaths during the study period of 1 January 2016 to 28 February 2021 by state in the United States.

Jurisdiction	Firearm-related incidents		Firearm-related non-fatal injuries		Firearm-related deaths	
	No.	No. per 100,000	No.	No. per 100,000	No.	No. per 100,000
Alabama	1,302	26.6	777	15.9	504	10.3
Alaska	225	30.6	65	8.8	59	8.0
Arizona	636	8.9	317	4.4	314	4.4
Arkansas	720	23.9	376	12.5	218	7.2
California	3,699	9.4	2,003	5.1	1,394	3.5
Colorado	781	13.7	351	6.2	225	3.9
Connecticut	785	22.0	357	10.0	85	2.4
Delaware	376	39.0	192	19.9	53	5.5
District of Columbia	688	98.1	428	60.9	135	19.2
Florida	3,430	16.1	1,704	8.0	1,027	4.8
Georgia	1,722	16.4	937	8.9	657	6.3
Hawaii	66	4.6	21	1.5	22	1.6
Idaho	139	8.0	37	2.1	42	2.4
Illinois	4,518	35.5	3,533	27.8	921	7.2
Indiana	1,456	21.7	815	12.2	433	6.5
Iowa	550	17.5	205	6.5	72	2.3
Kansas	535	18.4	260	8.9	143	4.9
Kentucky	1,030	23.1	529	11.9	291	6.5
Louisiana	1,864	40.0	1,158	24.8	572	12.3
Maine	163	12.2	33	2.5	27	2.0
Maryland	1,580	26.2	933	15.5	474	7.8
Massachusetts	1,306	19.0	403	5.9	118	1.7
Michigan	1,660	16.6	996	10.0	450	4.5
Minnesota	664	11.8	317	5.7	125	2.2
Mississippi	921	30.9	532	17.9	340	11.4
Missouri	1,626	26.6	945	15.4	609	9.9
Montana	127	12.0	34	3.2	39	3.7
Nebraska	408	21.2	157	8.1	57	2.9
Nevada	430	14.2	307	10.1	197	6.5
New Hampshire	187	13.8	33	2.4	23	1.7
New Jersey	1,196	13.5	670	7.5	252	2.8
New Mexico	402	19.2	150	7.1	149	7.1
New York	2,302	11.8	1,336	6.8	419	2.1
North Carolina	1,949	18.8	1,170	11.3	596	5.7
North Dakota	102	13.5	28	3.7	18	2.3
Ohio	2,559	21.9	1,500	12.8	682	5.8
Oklahoma	813	20.6	405	10.3	249	6.3
Oregon	470	11.2	186	4.4	110	2.6

© 2022 Sun S et al. *JAMA Network Open.*

Pennsylvania	2,652	20.7	1,757	13.7	658	5.1
Rhode Island	174	16.4	67	6.3	17	1.6
South Carolina	1,469	28.9	792	15.6	432	8.5
South Dakota	124	14.2	26	2.9	21	2.4
Tennessee	1,824	26.9	1,126	16.6	534	7.9
Texas	3,514	12.3	1,845	6.4	1,367	4.8
Utah	237	7.5	97	3.1	71	2.3
Vermont	80	12.7	14	2.2	14	2.2
Virginia	1,463	17.2	964	11.3	403	4.7
Washington	676	9.0	320	4.3	210	2.8
West Virginia	343	19.0	146	8.1	93	5.2
Wisconsin	1,163	20.0	638	11.0	220	3.8
Wyoming	45	7.8	11	1.9	17	2.9
United States	57,151	17.5	32,000	9.8	16,160	4.9

© 2022 Sun S et al. *JAMA Network Open.*

eTable 3. Percent excess firearm violence during the COVID-19 pandemic in the 50 US states and the District of Columbia using different spline functions and degrees of freedom (dfs) for the days from February 15, 2020, to February 28, 2021. eCI=empirical confidence interval.

Spline function	dfs	Firearm-related incidents (95% eCI)	Firearm-related injuries (95% eCI)	Firearm-related deaths (95% eCI)
Cubic B-spline	2 (Main model)	15.0 (4.6 to 26.1)	34.3 (26.1 to 41.1)	28.4 (12.9 to 46.2)
	3	15.3 (5.8 to 25.0)	34.6 (24.5 to 43.2)	28.5 (15.2 to 43.2)
	4	15.3 (5.9 to 24.9)	34.9 (20.5 to 49.2)	28.8 (13.4 to 44.4)
Natural cubic B-spline	3	15.6 (7.3 to 23.9)	36.2 (20.8 to 52.7)	27.2 (10.8 to 46.4)
	4	15.6 (7.4 to 23.9)	36.2 (20.8 to 52.7)	27.2 (10.8 to 46.4)
	5	15.6 (7.4 to 23.9)	36.2 (20.9 to 52.7)	27.2 (10.8 to 46.4)

© 2022 Sun S et al. *JAMA Network Open.*

eTable 4. Number of firearm violence and estimated excess number and percent excess during the coronavirus disease 2019 period (i.e., 1 March 2020 to 28 February 2021) in city of Philadelphia using data from Philadelphia Police Department and Gun Violence Archive (GVA). eCI=empirical confidence interval.

Characteristics	Philadelphia Police Department	Gun Violence Archive
Firearm-related incidents		
Total	2,324	1,997
Excess No. (95% eCI)	790 (635, 919)	264 (99, 407)
Excess % (95% eCI)	51.5 (37.6, 65.5)	15.2 (5.2, 25.6)
Firearm-related non-fatal injuries		
Total	2,410	2,004
Excess No. (95% eCI)	771 (611, 912)	241 (26, 423)
Excess % (95% eCI)	47.0 (34.0, 60.9)	13.7 (1.3, 26.8)
Firearm-related deaths		
Total	442	452
Excess No. (95% eCI)	89 (22, 138)	90 (21, 141)
Excess % (95% eCI)	25.0 (5.3, 45.4)	24.8 (5.0, 45.4)

© 2022 Sun S et al. *JAMA Network Open.*

eFigure 1. The number of firearm-related events per month per million people between January 1, 2018 and February 28, 2021 by state in the United States. COVID-19=coronavirus disease 2019.

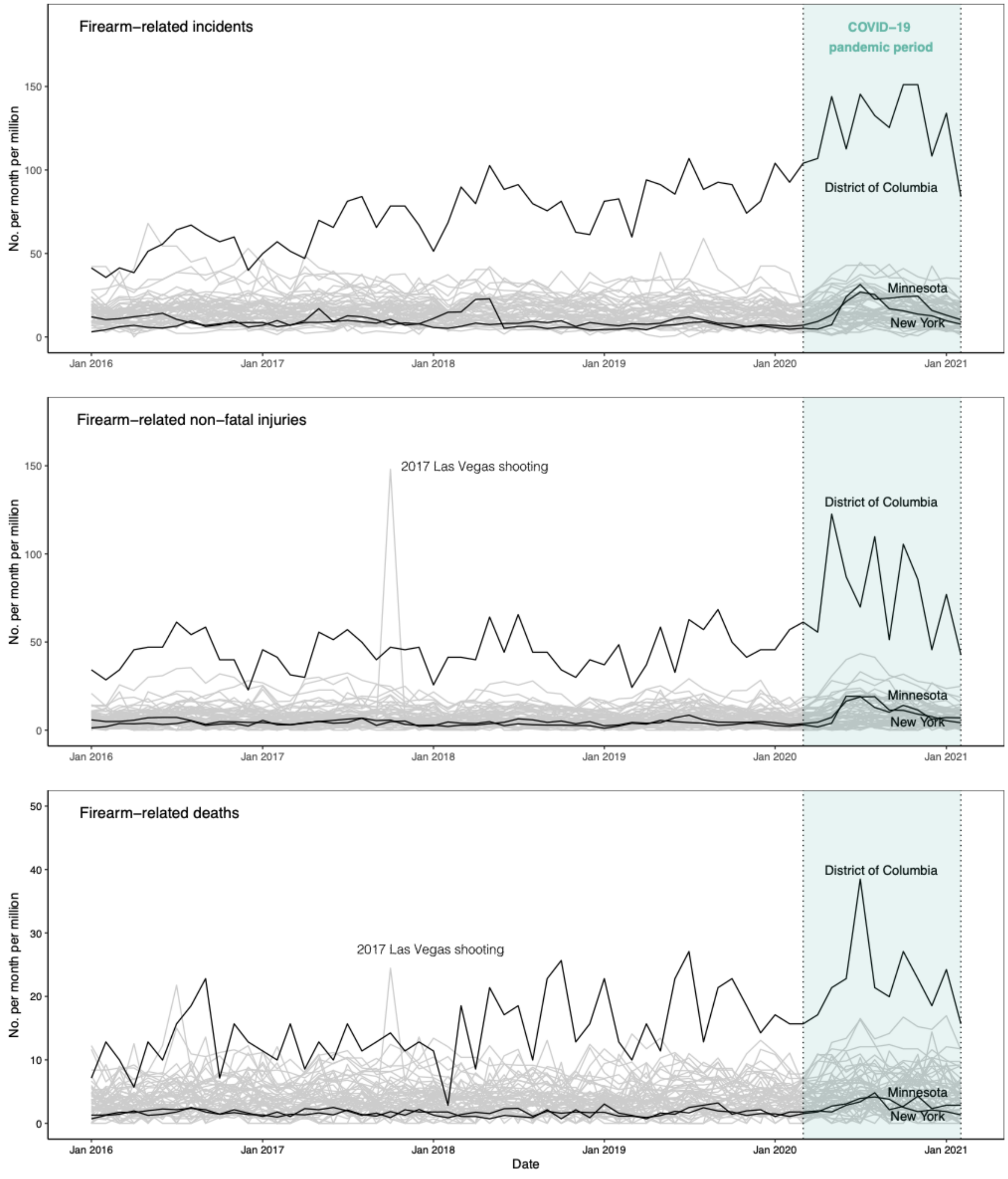

© 2022 Sun S et al. *JAMA Network Open.*

eFigure 2. Temporal trend in excess risk associated with the COVID-19 pandemic period using a quadratic B-spline (main model) and a natural spline with 3 degrees of freedom for the days from February 15, 2020, to February 28, 2021 in the United States.

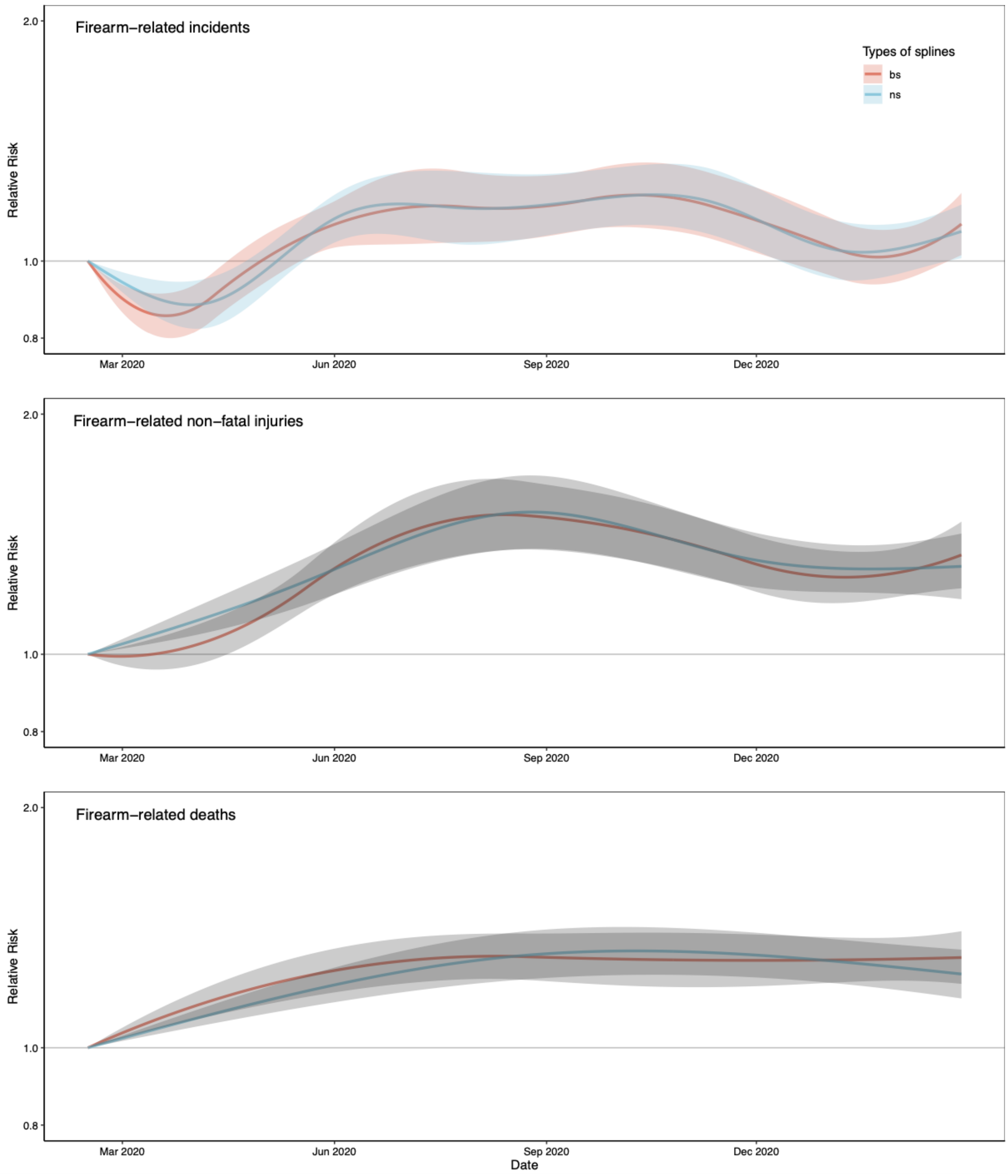

© 2022 Sun S et al. *JAMA Network Open.*

eFigure 3. Temporal trend in excess risk by state associated with the COVID-19 pandemic period by state in the United States.

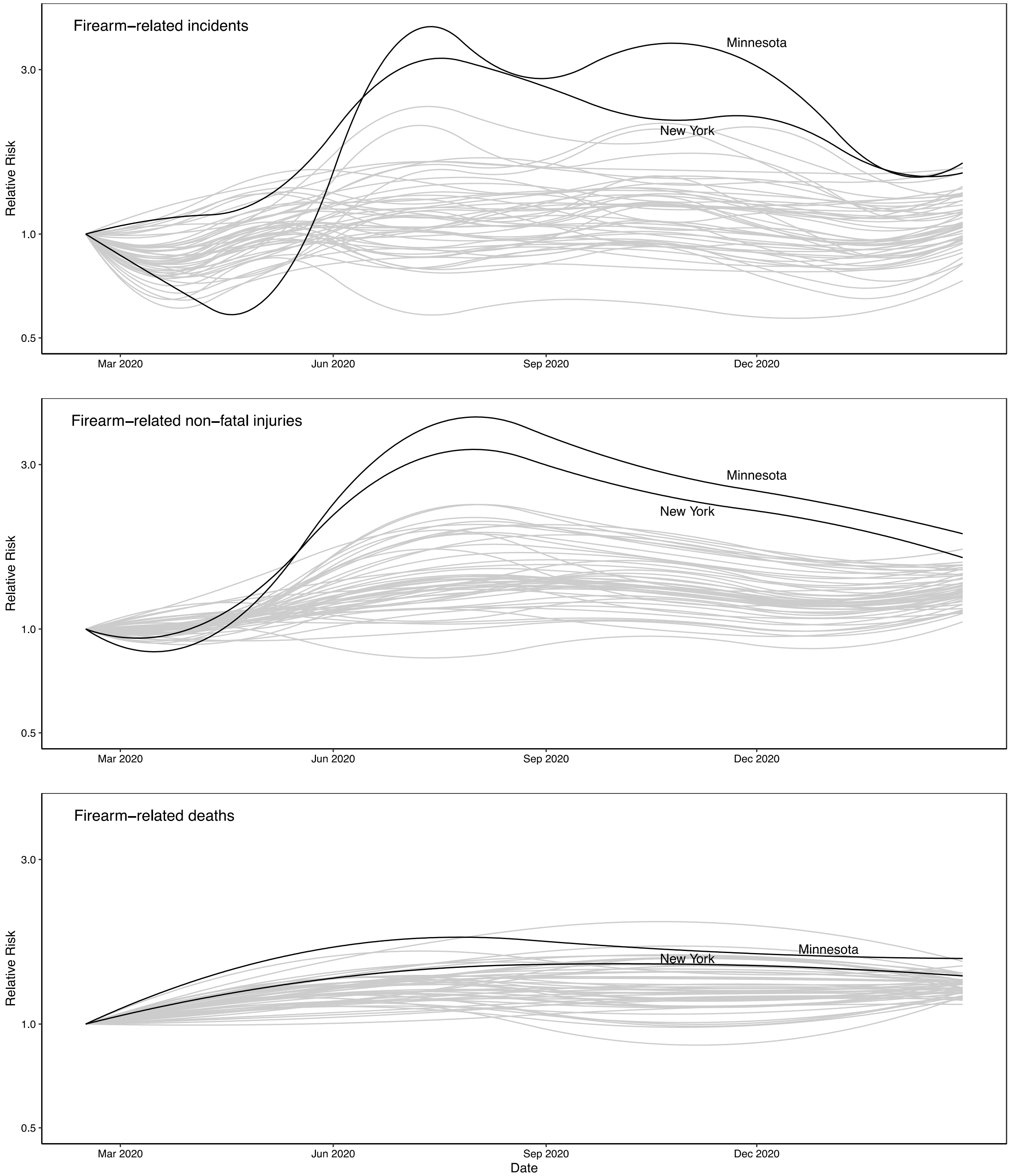

© 2022 Sun S et al. *JAMA Network Open.*

eFigure 4. The number difference of firearm-related events between data from Philadelphia Police Department and Gun Violence Archive (GVA). The positive number represents how many events were recorded in Philadelphia Police Department but not in GVA.

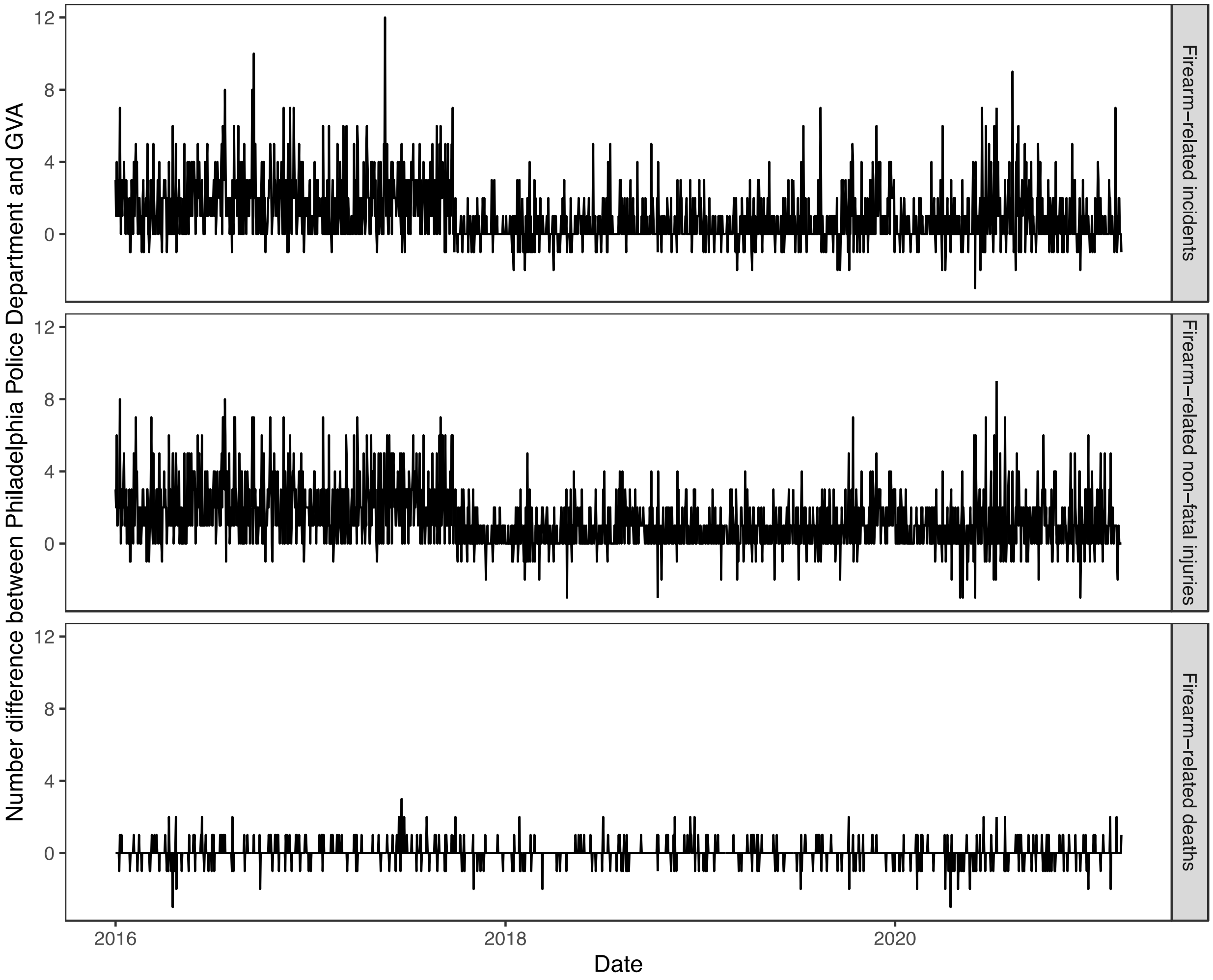

© 2022 Sun S et al. *JAMA Network Open.*

eFigure 5. The ratio of number of non-fatal to fatal incidents in GVA, January 1, 2016 to February 28, 2021.

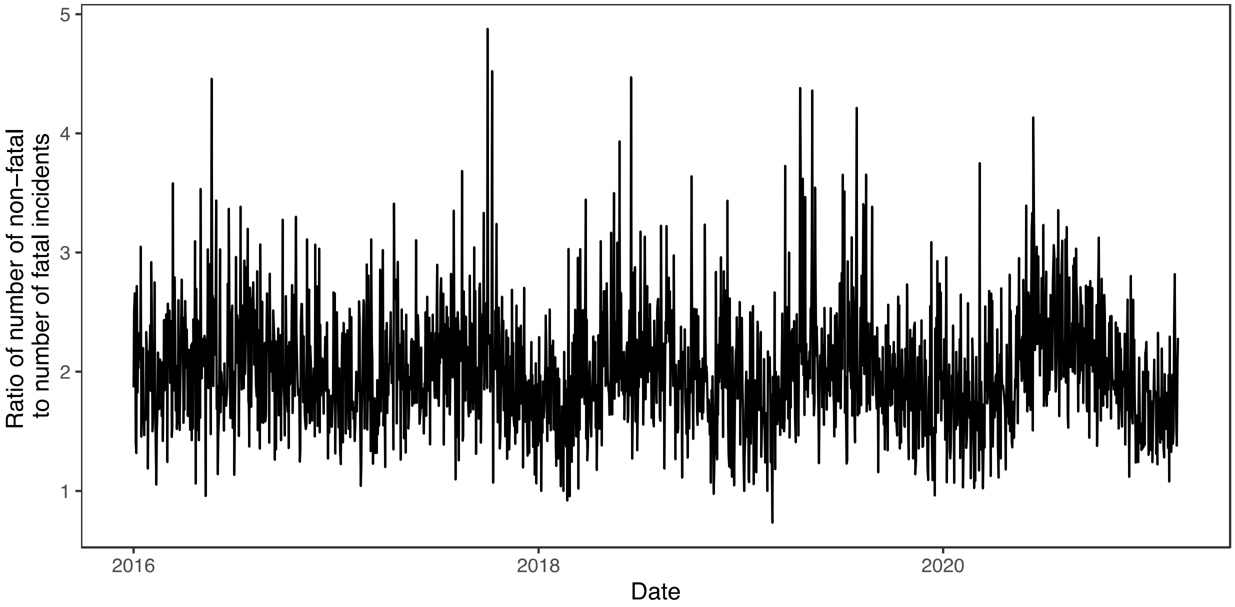

© 2022 Sun S et al. *JAMA Network Open.*

eFigure 6. Temporal trend in excess risk in firearm-related incidents (A), non-fatal injuries (B), and deaths (C) during the pandemic period in the city of Philadelphia using data from Gun Violence Archive (GVA) and Philadelphia Police Department (PPD). Each line represents the temporal trend in excess risk for each state. The band is the 95% confidence interval for the excess risk.

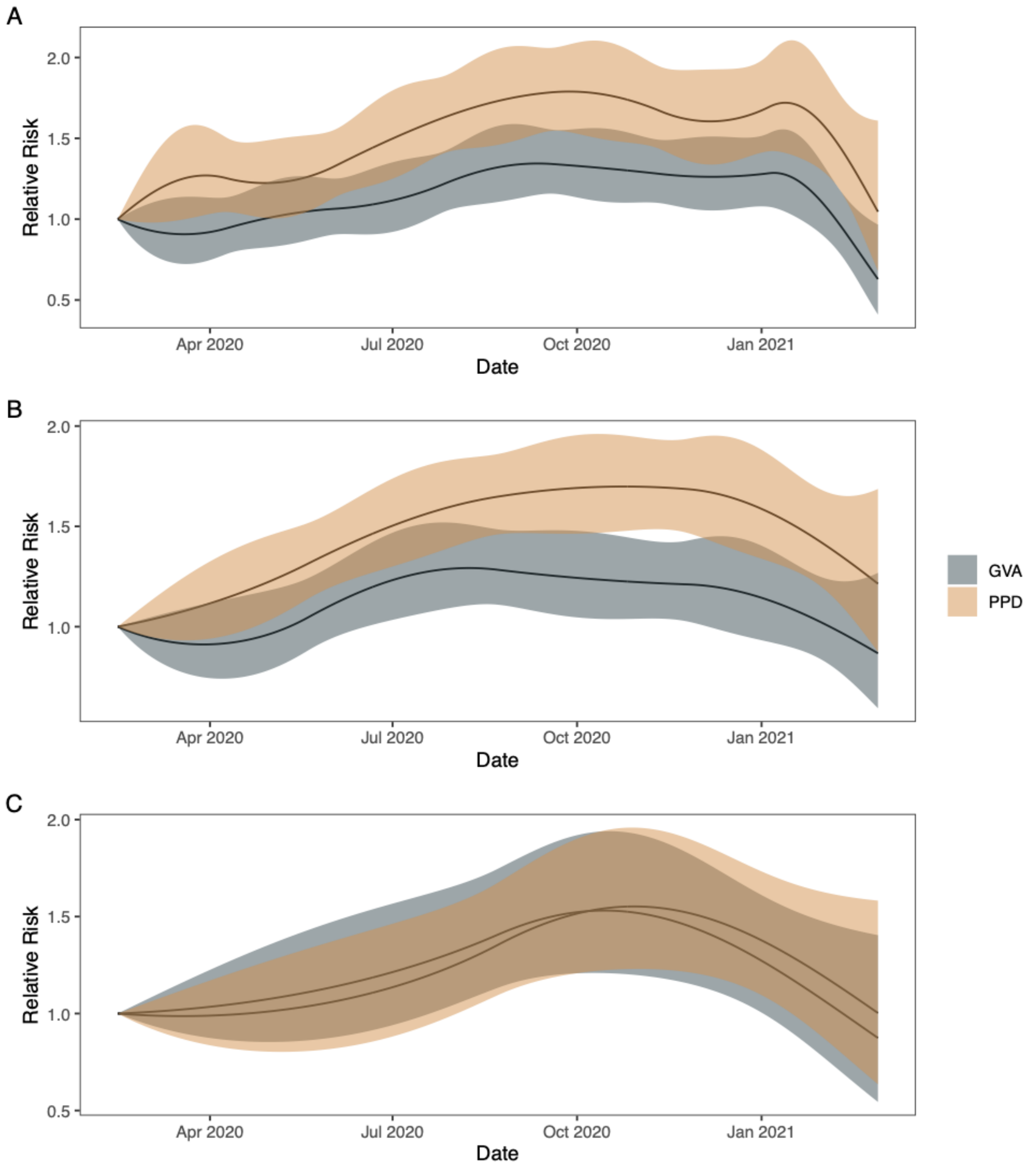

© 2022 Sun S et al. *JAMA Network Open.*

eReference

1. Johns Hopkins University Center for Systems Science and Engineering. COVID-19 Data Repository by the Center for Systems Science and Engineering (CSSE) at Johns Hopkins University. https://github.com/CSSEGISandData/COVID-19. Published 2021. Accessed May 11, 2021, 2021.
2. Boston University School of Public Health. COVID-19 US State Policies (CUSP). https://www.bu.edu/sph/news/articles/2021/new-website-tracks-compares-us-state-covid-policies/. Published 2021. Accessed May 11, 2021, 2021.
3. Michael Siegel. State firearm laws. https://www.statefirearmlaws.org/national-data. Published 2018. Accessed May 10, 2021, 2021.
4. FBI. NICS firearm checks: month/year by state/type. https://www.fbi.gov/file-repository/nics_firearm_checks_-_month_year_by_state_type.pdf/view. Published 2021. Accessed May 11, 2021, 2021.
5. US Bureau of Labor. Table 1. Civilian labor force and unemployment by state and selected area, seasonally adjusted. https://www.bls.gov/news.release/laus.t01.htm. Published 2021. Accessed May 10, 2021, 2021.
6. Siegel M, Pahn M, Xuan Z, et al. Firearm-related laws in all 50 US states, 1991–2016. *American Journal of Public Health (ajph)*. 2017.
7. Azad HA, Monuteaux MC, Rees CA, et al. Child access prevention firearm laws and firearm fatalities among children aged 0 to 14 years, 1991-2016. *JAMA pediatrics.* 2020;174(5):463-469.
8. Knopov A, Siegel M, Xuan Z, Rothman EF, Cronin SW, Hemenway D. The impact of state firearm laws on homicide rates among black and white populations in the United States, 1991–2016. *Health & social work.* 2019;44(4):232-240.
9. Raifman J, Nocka K, Jones D, et al. COVID-19 US state policy database. www.tinyurl.com/statepolicies. Published 2020. Accessed.
10. Sera F, Armstrong B, Tobias A, et al. How urban characteristics affect vulnerability to heat and cold: a multi-country analysis. *Int J Epidemiol.* 2019;48(4):1101-1112. doi:10.1093/ije/dyz008

© 2022 Sun S et al. *JAMA Network Open.*
